# Recent Ancestry of Kyasanur Forest Disease Virus

**DOI:** 10.3201/eid1509.080759

**Published:** 2009-09

**Authors:** Rajeev Mehla, Sandeep R.P. Kumar, Pragya Yadav, Pradip V. Barde, Prasanna N. Yergolkar, Bobbie R. Erickson, Serena A. Carroll, Akhilesh C. Mishra, Stuart T. Nichol, Devendra T. Mourya

**Affiliations:** National Institute of Virology, Pune, India (R. Mehla, S.R.P. Kumar, P. Yadav, P.V. Barde, P.N. Yergolkar, A.C. Mishra, D.T. Mourya); Centers for Disease Control and Prevention, Atlanta, Georgia, USA (B.R. Erickson, S.A. Carroll, S.T. Nichol)

**Keywords:** Kyasanur Forest disease virus, genotyping, RT-PCR, RNA polymerase, NS5 gene, evolution, Bayesian coalescent analysis, viruses, podcast, research

## Abstract

Kyasanur Forest disease virus (KFDV) is enzootic to India and maintained in ticks, mammals, and birds. It causes severe febrile illness in humans and was first recognized in 1957 associated with a high number of deaths among monkeys in Kyasanur Forest. Genetic analysis of 48 viruses isolated in India during 1957–2006 showed low diversity (1.2%). Bayesian coalescence analysis of these sequences and those of KFDVs from Saudi Arabia and the People’s Republic of China estimated that KFDVs have evolved at a mean rate of ≈6.4 × 10^–4^ substitutions/site/year, which is similar to rates estimated for mosquito-borne flaviviruses. KFDVs were estimated to have shared a common ancestor in ≈1942, fifteen years before identification of the disease in India. These data are consistent with the view that KFD represented a newly emerged disease when first recognized. Recent common ancestry of KFDVs from India and Saudi Arabia, despite their large geographic separation, indicates long-range movement of virus, possibly by birds.

## Abstract 2

Clinicians in Asia should consider this disease when diagnosing acute febrile illnesses.

Kyasanur Forest disease virus (KFDV) is a member of the mammalian tick-borne virus group (previously referred to as the tick-borne encephalitis serogroup) of the family *Flaviviridae* and genus *Flavivirus* (*1*). In addition to KFDV, this group contains Louping ill, tick-borne encephalitis, Omsk hemorrhagic fever, Langat, Powassan, Royal Farm, and Gadgets Gully viruses. KFD was first recognized in 1957 in the Kyasanur Forest of Shimoga District, Karnataka State, India, when a disease causing a high number of deaths was observed in 2 species of monkeys: the black-faced langur (*Semnopithecus entellus*, earlier known as *Presbytis entellus*) and the red-faced bonnet monkey (*Macaca radiata*).

Human cases were also found among persons who visited forests to collect firewood, grass, and other forest products. Human disease is characterized by an incubation period of ≈3–8 days, followed by chills, frontal headache, body ache, and high fever for 5–12 days, and a case-fatality rate >30% (*2*). During infection by KFDV, virus titer remains high <10 days after onset of symptoms, as reported by Bhat et al. (*3*). However, Upadhyaya et al. (*4*) found that viremia in patients lasted for 12–13 days of illness and unlike most other flaviviruses, remains high during the first 3–6 days with titers as high as 3.1 × 10^6^ PFU/mL.

Continuing deaths in monkeys and an average of 400–500 human cases have been seen annually over the past 5 decades, commonly occurring in evergreen, semi-evergreen, and neighboring, moist, deciduous forest areas. An array of tick species, mainly *Haemaphysalis spinigera*, act as vectors for KFDV (*5*). This species of tick is widely distributed in tropical evergreen and deciduous forests of southern and central India and Sri Lanka. KFDV has also been isolated from 7 other species of this genus and from *Dermacentor* and *Ixodes* ticks. This disease is transmitted by ticks among ground birds and small mammals such as the white-tailed rat, white-bellied rat, shrew, and bat. High titers of virus can be obtained after experimental infection of black-napped hares, porcupines, flying squirrels, Malabar giant squirrels, three-striped squirrels, gerbils, mice, long-tailed tree mice, and shrews (*2*–*9*).

Until 1971, KFDV was endemic to the Sagar, Sorab, and Shikaripur taluks (counties) of Shimoga District (Figure 1). By 1972, a new focus of virus activity appeared in Sirsi Taluk, Uttara Kannada District. Many KFDV isolates were obtained from Karnataka during 1957–1972 and maintained in a repository at the National Institute of Virology (NIV) in Pune, India. However, the virus was found to be highly infectious, as shown by numerous infections in field and laboratory personnel (*2*,*10*), which resulted in suspension of work with this virus until an appropriate BioSafety Level-3 laboratory was built at NIV in 2004. In 2006, this laboratory isolated a virus from a serum sample of a patient suspected of having KFD that was obtained from the Virus Diagnostic Laboratory in Shimoga.

**Figure 1:**
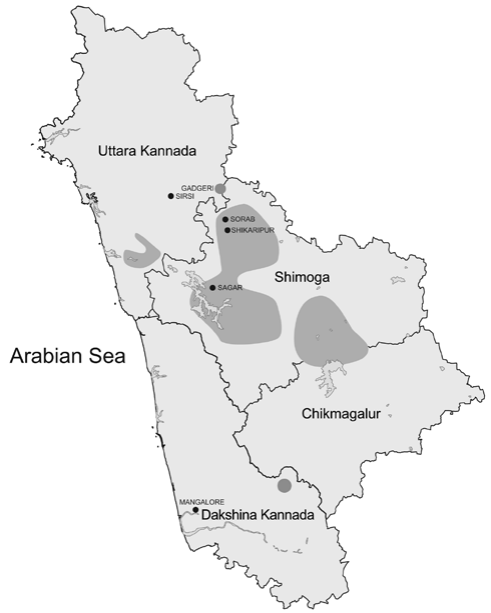
Areas of Karnataka State, India, known to be affected by Kyasanur Forest disease (dark gray shading).

More recent studies have identified KFDV in Saudi Arabia and the People’s Republic of China (*11*,*12*). During 1994–1995, a virus was isolated from hemorrhagic fever patients in the Makkah region of Saudi Arabia and identified as a KFDV variant, referred to as the Alkhurma variant or subgroup (*11*,*13*,*14*). The prototype strain of KFDV from Saudi Arabia (strain 1176, isolated in 1995) and the KFDV reference strain from India (P-9605, isolated in 1957) differ from each another by only 8% at the genome nucleotide level, despite their temporal (38 years) and geographic (≈4,000 km) separation. A virus initially referred to as Nanjianyin virus, isolated in 1989 from a febrile patient in Nanjian County in the Hengduan Mountain region of Yunnan Province in southwestern China, was recently identified as a strain of KFDV (*12*). However, it is unclear whether this KFDV 1989 isolate from China is an authentic virus isolate because it is virtually identical at the nucleotide level with the 1957 reference strain from India (P-9605), despite their being isolated 32 years and almost 3,000 km apart. The P-9605 strain was distributed widely to arbovirus reference laboratories. Reference KFDV virus was used as part of the analysis of serum samples from Yunnan Province (*15*,*16*).

Results of molecular epidemiologic studies have suggested that tick-borne flaviviruses have evolved slowly while dispersing north and west across Asian and European forests during the past few millennia (*17*–*19*). This pattern is different from that of rapidly evolving mosquito-borne flaviviruses, many of which can be transported long distances by migratory birds, persons, animals, or mosquito eggs (*19*,*20*). We examined the diversity and evolution of KFDV and present data that indicated that KFDV isolates from India, Saudi Arabia, and China share a recent common ancestor, indicating long-range movement of this tick-borne flavivirus. In addition, we also estimated the evolution rate of KFDV and compared it with that of mosquito-borne flaviviruses.

## Methods

### Virus Selection and Reverse Transcription–PCR

Forty-seven representative KFDV isolates from India were chosen for analysis; these isolates were obtained during 1957–1972 (Table 1). Isolates represented viruses from various host species and different geographic locations in Shimoga, Uttara Kannada, and Dakshina Kannada districts, Karnataka State. One KFDV from India isolated in 2006 was also included. Lyophilized KFDV stocks were obtained from the virus repository at the NIV, India, and grown in Vero E6 cell lines. Primers for PCR and phylogenetic analysis were designed to target regions of structural genes (premembrane/envelope) and the nonstructural protein 5 (NS5) gene (viral polymerase) (Table 2).

**Table 1:** Isolates of Kyasanur Forest disease virus analyzed, India*

ID no.	Isolate	Year	Location	Original source	Common name of source
1	W379	1957	Baragi	*Semnopithecus entellus*	Black-faced langur
2	P9605	1957	Shigga	*Homo sapiens*	Human
3	G11333	1957	Barasi	*Haemaphysalis spinigera*	Tick
4	P16011	1958	Kaisodi	*H. sapiens*	Human
5	W3399	1958	Hessare	*S. entellus*	Black-faced langur
6	W6043	1959	Belisiri	*S. entellus*	Black-faced langur
7	W6178	1959	Koppalgadde	*S. entellus*	Black-faced langur
8	G27667	1959	Kunvahalli	*Haemaphysalis spinigera* from dead monkey	Tick
9	P20924	1959	Mullukere	*H. sapiens*	Human
10	P21092	1959	Hadapsar	*H. sapiens*	Human
11	601203	1960	Tudikoppa	*H. sapiens*	Human
12	611661	1961	Sagar Station	*Haemaphysalis turturis*	Tick
13	612057	1961	Barur	*Rattus rattus wroughtoni*	White-bellied rat
14	62844	1962	Hillemarur	*H. spinigera*	Ticks
15	62849	1962	Hillemarur	*R. rattus wroughtoni*	White-bellied rat
16	62957	1962	Hillemarur	*H. sapiens*	Human
17	623969–2	1962	VRC Poona	*H. sapiens*	Human
18	63661	1963	Malvei	*H. sapiens*	Human
19	63696	1963	Suranagadde	*S. entellus*	Black-faced langur
20	64244	1964	Balagodu	*Ixodes petauristae*	Tick
21	64350	1964	Marasa	*Haemaphysalis formosensis*	Tick
22	642034	1964	Kangodu	*H. turturis*	Tick
23	642046	1964	Kangodu	*Haemaphysalis papuana kinneari*	Tick
24	652	1965	Kangodu	*Haemaphysalis wellingtoni*	Tick
25	651521	1965	VRC Poona	*H. sapiens*	Human
26	652980	1965	Vadnala	*Haemaphysalis spp.*	Tick
27	6616	1966	Yelagalale	*S. entellus*	Black-faced langur
28	66364–1	1966	VRC staff, Sagar	2-day acute-phase serum sample, *H. sapiens*	Human
29	66928–2	1966	Sagar	*H. sapiens*	Human
30	664518	1966	Kondagalale	*H. turturis*	Tick
31	67965	1967	Sagar	*H. sapiens*	Human
32	671004	1967	Bhadrapura	*S. entellus*	Black-faced langur
33	673514	1967	Siravala	*H. papuana kinneari*	Tick
34	68142	1968	Holagalale	*S. entellus*	Black-faced langur
35	68159	1968	Siravala	*H. turturis*	Tick
36	68484	1968	Halagalale	*Rattus blanfordi*	White-tailed wood rat
37	681960	1968	Barur	*H. sapiens*	Human
38	692156	1969	Chikkanallur	*H. spinigera*	Tick
39	692163	1969	Thonagodu	*H. sapiens*	Human
40	712419	1971	Nodahalli	*H. spinigera*	Tick
41	716810	1971	Gunjnur	*H. spinigera*	Tick
42	72166	1972	Gadgeri-sirsi	*Haemaphysalis kyasanurensis*	Tick
43	72827	1972	Holekoppa	*S. entellus*	Black-faced langur
44	A106	2006	Chikkanallur	*H. sapiens*	Human
45	W6204	1959	Kannahalli	*S. entellus*	Black-faced langur
46	G27678	1959	Kopalgadde	*H. spinigera*	Tick
47	W1930	1958	Chimnoor	*S. entellus*	Black-faced langur
48	601011	1960	Chikkasakuna	*H. sapiens*	Human

*ID, identification; VRC, Virus Research Centre.

**Table 2:** Primers used for diagnostic nested reverse transcription–PCR and genotyping of KFD virus, India*

Gene	Primer	Genome location	Primer sequence (5′ → 3′)	Product, bp	Type
preM–env	KFD-EF2	459–478	TGGTGTTCTCTGCGACAGTT	780	Genotyping
	KFD-ER2	1258–1238	TCTGTCACTCTGGTCTCGCTT		
	KFD-EF3†	606–628	TCATTCGAGTGTGTGTCACCATT		
	KFD-ER1†	701–678	TTCCGTATTCCAGTGACACTCGCT		
NS5	KFD-F3	9422–9441	GGCTGAGTCATGGACATCAT	642	
	KFD-R4	11046–11063	TCCACTCGTGTGGATGCT		
	KFD-F4†	9660–9680	TGAGACCTTCTGACGACCGCT		
	KFD-R3†	9801–9819	TCCTTCATCGTCAACTCAT		

*preM, premembrane; env, envelope; KFD, Kyasanur Forest disease; NS5, nonstructural protein 5. †Internal primers for sequencing some isolates.

Total RNA was extracted from 250 μL of infected Vero cell lysates by using Trizol reagent (GIBCO-BRL, Gaithersburg, MD, USA) per the manufacturer’s protocol. RNA was dissolved in 50 μL of nuclease-free water. cDNA was prepared separately for structural genes and NS5 by using avian Moloney virus reverse transcriptase (Promega, Madison, WI, USA). Briefly, 10 pM of each gene-specific reverse primer (ER2 and R4 were used for each set of the reverse transcription reactions, respectively) and incubated at 42°C for 45 min and then 85°C for 5 min.

cDNA was amplified by using 1U of Taq DNA Polymerase, 10× PCR buffer (Invitrogen. Carslbad, CA, USA), 0.2 mmol/L dNTP, 1.5 mmol/L MgCl_2_, and 10.0 pM of each primer pair as described in Table 2 in a reaction volume of 25 μL. PCR conditions included denaturation at 94°C for 5 min; 35 cycles of 1-min steps at 94°C, 55°C, and 72°C; and a 5-min extension at 72°C. Amplified products were analyzed by agarose gel electrophoresis. Bands of interest were recovered by using a DNA Gel Extraction Kit (QIAGEN, Valencia, CA, USA), according to the manufacturer’s protocol. Direct sequencing of the amplified product was conducted by using an ABI 3100 automated DNA sequencer and Big Dye terminator kit (Applied Biosystems, Foster City, CA, USA).

### Virus Sequence Analysis

The quality of each sequence was monitored by using Sequence Analysis software version 5.1 (Applied Biosystems). Sequences were assembled by using Kodon software version 2.1 (Appled Maths, Austin, TX, USA). Sequences were processed to give 720 nt of the structural gene (nt positions 500–1220) and 620 nt of the NS5 gene (nt positions 9440–10080) and submitted to the National Center for Biotechnology Information (Bethesda, MD, USA) NCBI (GenBank accession nos. EU293242–EU293289 and EU293290–EU293337, respectively). Multiple sequence alignments were generated by using the MAFFT function (*21*) in SeaView (*22*). Nucleotide and amino acid proportional distances were calculated and compared for each virus with their respective date of isolation.

A partition homogeneity test (*23*) was conducted by using PAUP* 4.0b10 (*24*) to demonstrate that it was not inappropriate to analyze the 720-nt structural gene fragment and 620-nt NS5 gene fragment as a colinearized or concatenated single sequence. Phylogenetic analysis was performed on the colinearized sequence from each of the 48 KFDV isolates from India (Table 1) along with the corresponding gene regions available in GenBank for additional KFDVs: a1989 KFDV isolate (Nanjianyin) reportedly from China (EU918174, NS5 and EU918175, polyprotein) and 2 KFDVs from Saudi Arabia isolated in 1995 (AF331718) and 2004 (DQ154114).

The Modeltest 3.7 software program (*25*) was used to examine 56 models of nucleotide substitution to determine the model most appropriate for Bayesian coalescent analysis of the KFDV dataset. The general time reversible evolutionary model incorporating invariant sites (GTR + I) was found to be the best fit to the data according to the Akaike information criterion. Bayesian phylogenetic analysis was conducted by using BEAST, BEAUTi, and Tracer analysis software (*26*) with the GTR + I model. Preliminary analyses were run for 10,000,000 generations to select the clock and demographic models most appropriate for the KFDV dataset. An analysis of the marginal likelihoods indicated that the relaxed lognormal molecular clock and constant population size model was decisively chosen (log_10_ Bayes factors of 3.113) for the KFDV dataset. Final data analysis included a Markov chain Monte Carlo chain length of 50,000,000 generations with sampling every 1,000 states.

## Results

Comparison of nucleotide sequences of colinearized fragments of structural (720 nt) and NS5 (620 nt) genes of 48 KFDV isolates from India collected over the past 5 decades (Table 1) showed a low level of diversity among these viruses (GenBank accession nos. EU29242–EU29337). A maximum of 1.2% nt and 0.5% aa differences were seen among these viruses; the most divergent virus was the A106 virus isolated in 2006. Most viruses were isolated during 1957–1972. That the 2006 virus isolate is the most divergent is consistent with the 34-year gap in sampling. As expected, little diversity was seen among the virus isolates irrespective of the host, which included humans, black-faced langurs, red-faced bonnet monkeys, various tick species (*H*. *spinigera*, *H*. *kyasanurensis*, *H*. *turturis*, *H*. *papuana kinneari*, *H*. *wellingtoni*, *H*. *formosensis*, and *Ixodes petauristae*), and rodents (*Rattus rattus wroghtoni* and *R*. *blanfordi*) (Table 1). The sequence of the 1957 KFDV reference strain (P9605) from India and strain 651521 isolated from an NIV laboratory staff member in 1965 were identical, despite their 8-year separation. However, the staff member was accidently infected while handling reference KFDV, which provided an explanation for this anomaly.

All KFDV isolates from India differed from the Alkhurma variant of KFDV (*27*) found in Saudi Arabia by ≈8%–9% at the nucleotide level. This finding is similar to the extent of diversity (8%) reported in a comparison of the complete genome of a KFDV isolate from India with that of an isolate from Saudi Arabia (*28*). In contrast, the 1989 KFDV isolate (Nanjianyin) reportedly from China (*12*), differed by only 1 nt (1/1,320 [0.08%]) from the 1957 KFDV reference strain (P9605) from India and the laboratory infection strain 651521. It is notable that of the 48 KFDV strains from India analyzed, the KFDV strain from China should be most similar to strain P9605, a reference strain that was distributed worldwide to arbovirus reference laboratories. The KFDV 1989 isolate from China is virtually identical at the nucleotide level to the 1957 reference strain (P9605) from India, despite their being isolated 32 years and almost 3,000 km apart, which suggests that the strain from China is not an authentic virus isolate. A reference KFDV from India appears to have been used in the analysis of serum samples from Yunnan Province (*15*,*16*), which suggests a potential source of laboratory cross-contamination. In addition, the 2 sequence fragments (EU918174 for NS5 and EU918175 for the polyprotein) of the KFDV isolate reportedly from China appear to contain several sequence analysis errors; neither fragment encodes a functional protein because of creation of a stop codon and 2 frame shifts relative to KFDV reference sequences (AY323490 and EU480689).

**Figure 2:**
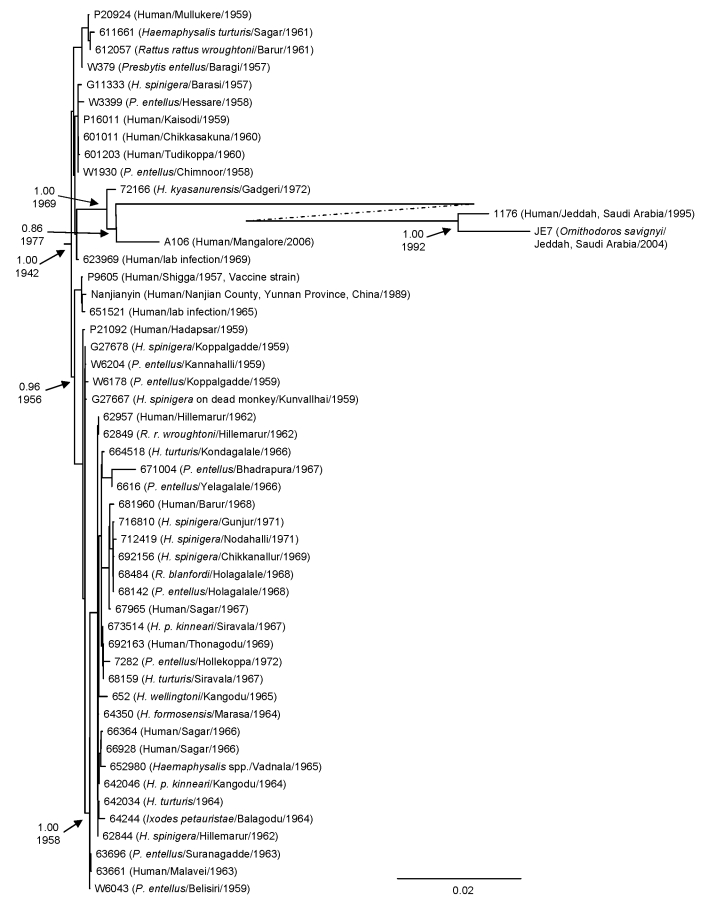
Bayesian coalescent analysis of sequence differences of Kyasanur Forest disease virus isolates from India (1957–2006), People’s Republic of China (1989), and Saudi Arabia (1995–2004). Analysis was conducted by using the general time reversible model incorporating invariant sites, a relaxed molecular clock, constant population size, and the BEAST, BEAUTi, and Tracer analysis software (*26*). The maximum clade credibility tree is depicted. Posterior probability values are indicated for clades of interest with the time to most recent common ancestor shown below. Scale bar indicates nucleotide substitutions per site.

Bayesian coalescent analysis of sequence differences among the 48 KFDV isolates from India (1957–2006), the isolates from Saudi Arabia (1995–2004) (*28*,*29*), and the reported isolate from China (1989) (*12*) was conducted to estimate the rate of evolution and time to the most recent common ancestor (MRCA) for these viruses (Figure 2). These viruses were estimated to be evolving at a mean rate of 6.4 × 10^–4^ substitutions/site/year (95% highest probable density [HPD] 4.1–8.8 × 10^–4^ substitutions/site/year). This estimate is similar to rates for other flaviviruses analyzed by using similar Bayesian coalescent methods, including a rate of 2.17 × 10^–4^ substitutions/site/year obtained for 23 St. Louis encephalitis viruses collected during 1933–2001 (*30*) and a rate of 4.2 × 10^–4^ substitutions/site/year for yellow fever virus (*31*). The finding of similar evolutionary rates for tick-borne and mosquito-borne flaviviruses was unexpected, given earlier assertions that evolution of tick-borne viruses was more gradual than rapidly evolving mosquito-borne viruses (*19*).

Analysis estimated that the mean time to the MRCA for all the KFDV isolates was only 64 years (95% HPD 51–84 years) before 2006 (the year when the most contemporary virus was isolated). The analysis estimated that these viruses shared a common ancestor as recently as ≈1942. Analysis of only KFDV isolates from India provides a slightly more recent estimate of their MRCA (≈1948), just 9 years before identification of the disease in Kyasanur Forest in 1957. This finding correlates well with the perception of local villagers and healthcare providers in the Kyasanur Forest area that this was a newly emerged disease (*32*). Massive deaths of monkeys or compatible human disease in the region were not reported before the 1957 disease outbreak. In the initial years, disease activity was reported in a limited area of ≈100 km^2^ in Sagar and Sorab taluks of Shimoga District. However, after 1972, epizootics and epidemics were recognized in several new foci, increasingly more distant from the original focus.

## Discussion

Most viruses analyzed were isolated in various small hamlets from migrating persons within the early enzootic zone in the Shimoga District (until 1972). Attempts to examine the relationship between genetic differences in a virus isolate relative to geographic location did not show any notable findings because of small differences and distances involved. However, the 1972 virus 72166 was isolated from a tick in the village of Gadgeri in Sirsi (Uttara Kannada District), which is north of Shimoga District. The 2006 virus A106 was isolated from a person south of Shimoga District, in Mangalore (Dakshina Kannada District) and further from the original virus epicenter (Figure 1). Although much of the topology of the virus phylogenetic tree generated by Bayesian coalescent analysis is poorly supported (nodal support posterior probability values <95), there is support for a branch that contains the 72166 1972 and the A106 2006 virus isolates (Figure 2). These data suggest that there may be an association between virus genetic divergence and temporal and geographic spread of KFDV in Karnataka, consistent with the concept of virus spreading over time from an initial focus of activity. Why this initial focus of virus activity occurred in this location and at this time remains unclear, but speculation includes emergence of the virus from a cryptic forest cycle caused by changes in land use or introduction of the virus from elsewhere by birds. A more complete picture should emerge with analysis of additional virus samples (particularly from the post-1973 period) and complete virus genomes.

Bayesian analysis estimates that the 1995 and 2004 KFDV isolates from Saudi Arabia shared a common ancestor in 1992. The node connecting these viruses with the 2006 KFDV isolate from India was in 1977, and a strongly supported node (1.0) shows that the 1972 and 2006 KFDV isolates from India shared a common ancestor with the viruses from Saudi Arabia in ≈1969. The simplest interpretation of these data and the epidemiologic observations would be that KFDV was introduced from India into Saudi Arabia in the late 1970s or the 1980s.

Similar findings of low genetic diversity and recent common ancestry were reported for the KFDVs from Saudi Arabia in a more limited study of 11 virus isolates collected over a 5-year period (1994–1999). Only 0.4%, 0.6%, and 0.9% genetic diversity were found in the E, NS3, and NS5 gene fragments of these isolates, respectively (*27*). Using these gene fragments along with those of the complete genome sequence of KFDV from India, the authors estimated divergence time by using an older method based on distance analysis of nonsynonymous substitutions. This estimate indicated recent ancestry of these viruses. The KFDV strains from Saudi Arabia were estimated to have diverged from one another over a 4–72 year period and the KFDVs from India and Saudi Arabia were estimated to have diverged 66–177 years ago (*27*).

It is unclear what factors influenced the apparent emergence of KFDV in Shimoga District, India, in 1957 and in the Makkah/Jeddah region in Saudi Arabia in 1994. Also unknown is how this tick-borne virus moved over the large distance between these regions. A considerable amount of knowledge has been accumulated with regard to the ecology of KFDV in India (*32*). The natural history of the virus is complex and involves dynamic cycles of various life stages of Ixodid ticks (primarily *H*. *spinigera*, but also other *Haemaphysalis* spp. ticks and *Ixodes* ticks) and amplifying (vertebrate) hosts, including rodents and shrews, and possibly monkeys and cattle. Increased human populations in the Sagar and Sorab taluks in the early 1950s may have been the primary catalyst for emergence of KFD in 1957. During 1951–1961, the population of Sagar Taluk increased 116%, bringing with it increases in deforestation, cattle grazing, and extension of paddy fields and cleared grazing areas deeper into previously forested areas (*32*). Expansion of the cattle population may have been a crucial factor because cattle harbor adult forms of *H*. *spinigera* ticks, and an association between cattle and increases in tick larval density has been described (*32*). Cattle also carry all life stages of other *Haemaphysalis* spp. ticks, which have been shown to be infected with KFDV. Thus, cattle would likely increase tick densities in cleared forest areas most frequented by humans. In addition, rats, shrews, and mice are highly susceptible to KFDV infection, and numerous virus isolates have been obtained from organs of infected animals (*33*). Changes in land use and population densities may have resulted in emergence of KFDV from a cryptic enzootic cycle in this previously heavily forested area.

A high percentage of birds in the affected area are positive for antibodies reactive with KFDV and infested with *Haemaphysalis* spp. and other tick genera, particularly larvae and nymphs (*32*). It is unclear whether birds play a role in the complex virus maintenance cycle in an enzootic zone, but birds carrying virus-infected ticks or migration of viremic birds could spread KFDV over large distances such as those separating areas of KFDV activity in India and Saudi Arabia (*19*,*34*). There is serologic evidence of KFDV, or a related flavivirus in the mammalian tick-borne virus group, in Saurashtra, Gujarat State, on the coast of India on the Arabian Sea and in birds captured outside Karnataka State (*2*,*10*,*32*).

The current known distribution of KFDV is limited to relatively restricted areas of India and Saudi Arabia. However, it is likely that the virus exists in other areas in cryptic enzootic cycles or is associated with unrecognized or undiagnosed disease. This finding, together with the distance separating the KFDV-affected areas in India and Saudi Arabia, despite their relatively recent common ancestry, suggests that KFD has the potential to flare up in other regions because of virus movement or ecologic changes in the area. Clinicians should consider KFD in a differential diagnosis when considering acute febrile cases with compatible symptoms in other regions of Asia and the Middle East.
